# Genome and Transcriptome Analysis of the Food-Yeast *Candida utilis*

**DOI:** 10.1371/journal.pone.0037226

**Published:** 2012-05-18

**Authors:** Yasuyuki Tomita, Kazuho Ikeo, Hideyuki Tamakawa, Takashi Gojobori, Shigehito Ikushima

**Affiliations:** 1 Central Laboratories for Frontier Technology, KIRIN Holdings Company, Ltd., Yokohama, Kanagawa, Japan; 2 Center for Information Biology and DNA Data Bank of Japan, National Institute of Genetics, Mishima, Shizuoka, Japan; The University of Nottingham, United Kingdom

## Abstract

The industrially important food-yeast *Candida utilis* is a Crabtree effect-negative yeast used to produce valuable chemicals and recombinant proteins. In the present study, we conducted whole genome sequencing and phylogenetic analysis of *C. utilis*, which showed that this yeast diverged long before the formation of the CUG and *Saccharomyces*/*Kluyveromyces* clades. In addition, we performed comparative genome and transcriptome analyses using next-generation sequencing, which resulted in the identification of genes important for characteristic phenotypes of *C. utilis* such as those involved in nitrate assimilation, in addition to the gene encoding the functional hexose transporter. We also found that an antisense transcript of the alcohol dehydrogenase gene, which *in silico* analysis did not predict to be a functional gene, was transcribed in the stationary-phase, suggesting a novel system of repression of ethanol production. These findings should facilitate the development of more sophisticated systems for the production of useful reagents using *C. utilis*.

## Introduction

*Candida utilis* (*Lindnera jadinii*) is a Crabtree effect-negative yeast that is currently used to produce several industrially important compounds, such as glutathione and RNA [1]–[3]. *C. utilis* can grow on inexpensive substrates, such as pulping-waste liquors from the paper industry [4], and high cell density culture and large-scale production are possible under efficient continuous-culture conditions [1]. Furthermore, *C. utilis* is able to assimilate nitrate, a naturally occurring mineral source of nitrogen found as potassium nitrate. Along with *Saccharomyces cerevisiae* and *Kluyveromyces fragilis*, dried *C. utilis* cells have been approved for use as a food additive by the Food and Drug Administration [1]. In addition, since efficient methods of *C. utilis* transformation have been developed [5]–[7], the yeast has been used for the heterologous production of monellin, α-amylase, carotenoids, and organic acids such as L-lactic acid [8]–[12].

In a previous study, *C. utilis* NBRC0988 (ATCC 9950, CBS 5609) was suggested to be tetraploid based on multiple gene disruption using the Cre-*loxP* system and flow-cytometric analysis of nuclear DNA content [7]. Tetraploidy is relatively uncommon, as most yeast do not maintain a stable ploidy level of greater than two [13]. The recent completion of the *C. utilis* genome by massively parallel sequencing has led to the identification of 37 potential secretome proteins and the conclusion that *C. utilis* is not a member of the CUG clade, which comprises species such as *Candida albicans*, *Debaromyces hansenii*, and *Pichia stipitis* [14]. To date, phylogenic analyses have been conducted based on ribosomal DNA sequences [14]–[16], and not on whole genome data. In addition, the whole transcriptome analysis of *C. utilis* has not yet been reported.

We first sequenced the entire genome of the *C. utilis* NBRC0988 strain and analyzed its transcriptome. Using these data, we then determined both its phylogenic position, and the relationship between its phenotype and its genetic background. In addition, we identify a putative novel genetic mechanism of regulation of ethanol production based on comparative genome and transcriptome analyses using the next-generation sequencing.

## Results

### Genome sequencing, assembly and accuracy

We first subjected *Candida utilis* NBRC0988 to whole genome sequence analysis. employed a hybrid approach to integrate Sanger and 454/Roche sequencing (GS-FLX) data, leading to the production of 975 supercontigs (Materials and Methods). supercontigs out of 975 were larger than 40-kb (kilobases) in length, and we defined the 13 supercontigs as chromosomes with a total estimated size of 12.8 Mb., the other supercontigs were organized into a single hypothetical chromosome referred to as unmapped chromosome (Table 1) (estimated total genome size: 14.6 Mb) (accession numbers BAEL01000001-BAEL01001163 and DG000065-DG000077). The nuclear DNA content of *C. utilis* NBRC0988 was 3- to 5-fold greater than that of a *S. cerevisiae* haploid strain (12.3 Mb), supporting our previous suggestion that the former is tetraploid [7].

**Table 1:** Genome sequencing overview.

Genome size (Mb)	14.6
Assembled chromosome	13
Unmapped chromosome	1
Genome GC content (%)	45.36
Total of genes	8,864
Coding genes	8,646
Coding (%)	59.2
Coding GC content (%)	45.35
tRNA genes	191
rRNA genes	27

The sequencing reads produced by the GS-FLX pyrosequencers are prone to nucleotide over-calls and under-calls that eventually manifest as insertion and deletion errors particularly within a homopolymer region (for example, poly-A) [17], [18]. calling errors may lead to two types of annotation errors: interrupted open reading frames (ORFs), with less than a 600-bp (base pair) gap between two ORFs, or overlapping ORFs. The interrupted and overlapping ORFs represented 6.3% (296) and 1.1% (52) of the total 4,706 annotated genes with at least one homolog in reference database mentioned below, respectively (frame-shift errors). Any doubtful assembly was considered to arise due to frame-shifts errors that resulted in two ORFs being annotated with the same name without having a truly orthologous relationship with each other (BLASTP e-value of more than 1e-5).

### Annotation of the *C. utilis* genome

Protein coding genes were predicted using GlimmerM [19] and GeneLook [20] for the 975 supercontigs (Materials and Methods; Figure S1). This resulted in the identification of a total of 8,646 protein coding genes of which 4,041 (46.7%) had at least one homolog (with a BLASTP e-value of 1e-10 and the prescribed annotation accuracy in Figure S2) in the *Saccharomyces* genome database (SGD), *Candida* Genome Database (CGD), and/or SwissProt release 55.6. Thus, the protein coding genes occupy 59.2% of the genome sequence (Table 1). In addition, each putative 8,864 genes in *C. utilis* was compared with *S. cerevisiae* S288C mitochondrial genome (85,779 bp) to reveal that nine orthologous genes in the unmapped chromosome were identified as mitochondrial genes (e-values were each less than 1e-10). Although Buerth et al. [14] predicted only a putative 6,417 ORFs, our RNA-Seq data shown below supported the existence of approximately 90% of our 8,864 predicted genes based on the detection at least once in RNA-Seq tags described in fourth section.

Protein functions were assigned to 40.2% (3,476 genes) of the predicted 8,646 genes according to the Eukaryotic orthologous groups of proteins (KOG) classification [21] (Figure S3). secretion signal peptide was predicted in approximately 9% (770 genes) of the total number of genes with PrediSi [22], and 4,048 of the predicted proteins contained Pfam domains. Comparison of protein domains with those present in nine other yeast*s* species (*Saccharomyces cerevisiae* [23], *Schizosaccharomyces pombe* [24], *Pichia stipitis* [25], *Yarrowia lipolytica* [26], *Ashbya gossypii* [27], *Kluyveromyces lactis* [26], *Candida albicans* [28], *Candida glabrata* [26], and *Debaryomyces hansenii* [26]), identified 23 functional domains in 24 genes that are specific to *C. utillis* (Table S1).

### The phylogenic position of *C. utilis*

**Figure 1:**
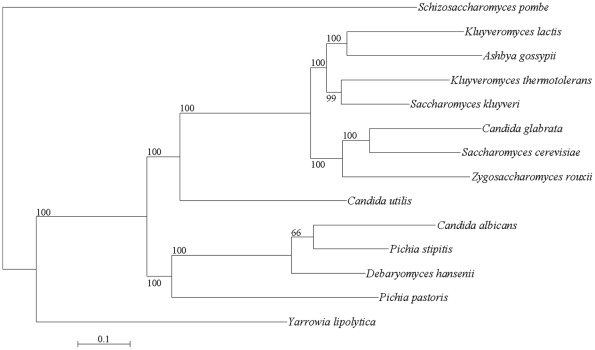
Phylogenetic tree of single copy orthologous genes in 14 species. This tree was built on the concatenated sequence of 32 single copy orthologous genes common to all 14 species by the maximum likelihood (ML) method using RAxML 7.2.8 with a JTT + Γ (gamma) model [58]. Individual orthologous gene families were aligned with the MUSCLE program [56], [57] and concatenated together to yield an alignment of 19,371 amino acid sites with gaps. One thousand bootstrap replicates were performed resulting in a fully resolved tree with all but one node having bootstrap values of 100.

We have first reported the phylogenic position of *C. utilis* using whole genome sequencing data. The phylogenic tree (Materials and Methods; Figure 1) shows that *C. utilis* diverged long before the formation of the CUG or *Saccharomyces*/*Kluyveromyces* clades. The codon usage of *C. utilis* differs significantly from that of other yeasts such as *S. cerevisiae*. For example, the relative frequencies of the usage of the two codons for Phe (UUU and UUC) and Tyr (UAU and UAC) are reversed in *C. utilis* relative to their frequencies of usage in *S. cerevisiae* (Figure S4).

### Analysis of the *C. utilis* transcriptome

A summary of the RNA-Seq data obtained using the next-generation sequencer is shown in Table 2. We obtained a total of 117,764,576 RNA-Seq tags from a log-phase sample in the presence of D-glucose and a total of 152,414,537 tags from a stationary-phase sample following depletion of D-glucose (accession number DRA000498). A majority of the expressed RNAs from each sample (approximately 60%) were successfully aligned to *C. utilis* genome (Table 2; Materials and Methods), resulting in the detection of more than 90% of the predicted genes based on detection of their transcription under the given conditions. In addition, more than 70% of the 3,186 genes that did not correspond to any protein in the reference database (predicted protein in Figure S2) were identified at least once in RNA prepared from cells at either phase. In this study, the RNA-Seq data were normalized based on upper-quartile (uq; defined by Bullard *et al.* [29]) and the gene whose uq value was more than 10 (approximately top 2 percent) was considered as “high expression gene (HEG)”. We then identified the pathways in which more than 10% of enzymatic genes were classified into HEGs either in log- (Table S3) or stationary-phase (Table S4). The numbers of the HEGs at log-phase, such as aminoacyl-tRNA and steroid biosyntheses genes, were notably more than that of stationary-phase, whereas citrate cycle and glyoxylate and dicarboxylate metabolism were more activated at stationary-phase. Besides, the HEGs at both phases were identified in oxidative phosphorylation pathway.

**Table 2:** RNA-seq overview.

Contents	Log-phase (10.5 h)	Stationary-phase (24 h)
Reference bases	14,496,988	14,496,988
Number of reads	117,764,576	152,414,537
Aligned to genome	71,588,205 (60.8%)	88,243,942 (57.9%)
Uniquely aligned to genome	65,389,686 (55.5%)	80,917,688 (53.1%)
Number of contigs	62,530	57,250
Total length of contigs	14,060,980	14,009,028
Number of SNPs	51,788	46,098
Reference covered	97.0% / 200%	96.6% / 200%
Non-coding (>180 bp)	4,031	4,091
Intergenic region	1,825	1,750
Antisense region	2,206	2,341

RNA-Seq analyses also revealed that approximately 97% of the genome covering both strands was expressed as RNA-Seq tags within each sample (theoretical maximum was 200%). Transcripts that were mapped to locations distinct from predicted exons may represent extensions of existing transcripts, non-coding RNA (ncRNA), antisense transcripts, or simply biological or experimental background errors. particular, we searched for transcripts within intergenic regions and antisense regions of coding sequences by identifying more than 180-bp contigs with a significant level of expression. Of 4,031 expressed regions identified at log-phase, 1,825 and 2,206 contigs were expressed in intergenic or antisense regions, respectively. Similarly, 1,750 and 2,341 contigs were found within intergenic or antisense regions during the stationary-phase (Table 2).

### Construction and comparison of protein families

Comparison of the amino acid sequences of *C. utilis* ORFs with those of the aforementioned nine yeasts and *Pichia pastoris* [30] was conducted using a tBLASTN search (e-value 1e-10). Thirty-five annotated proteins lacked homologous sequences (defined as “unique”) within these genomes and three proteins also lacked annotation in the other yeast genomes (defined as “specific”) (Table S2).

Next, the proteins were subsequently classified into families, using the protein sequences from the 10 yeasts (Materials and Methods). 36.4% (3,144) of the predicted coding genes of *C. utilis* belonged to a protein family common to at least one other yeast. We show venn diagram groupings of protein families in *C. utilis*, *S. cerevisiae*, and *C. albicans* in Figure S5, as the two yeast genomes were used for the annotation of *C. utilis* genes. a result, we identified 4,894 families that were unique to *C. utilis*. is, the 4,894 families lacked homologous sequences in *S. cerevisiae* and *C. albicans*. In addition, we identified 40 pathways in which either *S. cerevisiae* or *C. albicans*, or both, lack one or more of the genes in *C. utilis* defined in the KEGG database (Table S5).

For example, the *C. utilis* nitrate reductase (EC 1.7.1.1/2/3) (*CuNR*) does not belong to a protein family common to the other two yeasts, and a nitrite reductase (EC 1.7.1.4) was found in *C. utilis* (*CuNIR*) but not in the others. Taken together with the existence of a nitrate/nitrite transporter (*CuNRT*) specific to *C. utilis* (Table S2), these results are consistent with the existence of a nitrate/nitrite assimilation phenotype [31] that is unique to *C. utilis* among the yeasts analyzed.

### A cluster of nitrate assimilation genes in *C. utilis*

**Figure 2:**
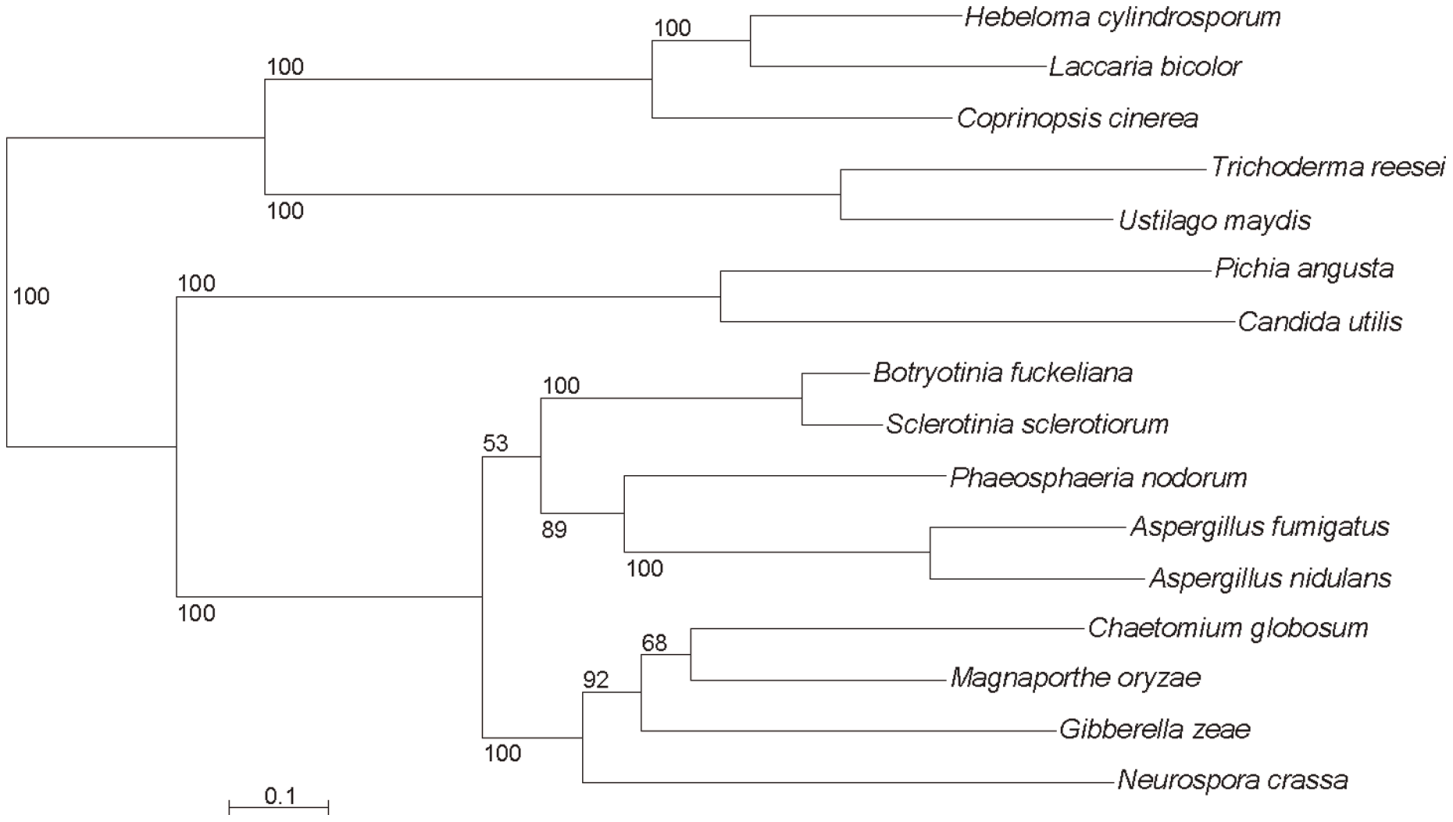
Phylogenetic tree of nitrate assimilation related genes. This tree was built on concatenated homologous sequences of all three genes (nitrate/nitrite transporter, nitrate reductases, and nitrite reductase) in Ascomycota and Basidiomycota from the subkingdom Dikarya (Materials and Methods) by the ML method.

The cluster of nitrate/nitrite assimilation genes was identified in various organisms which can assimilate nitrate and nitrite [32]. Our *C. utilis* genome sequence revealed an existence of contiguous nitrate/nitrite assimilation gene cluster located on chromosome 2. The gene cluster is also considered to be responsible for in the nitrate assimilation phenotype in *C. utilis*. Since some reports have suggested that this gene cluster was transferred horizontally from a Basidiomycete (mushrooms and smuts) to an ancestor of the Ascomycetous mold *Trichoderma reesei* [32], we performed concatenated phylogenetic analyses of homologs of all three genes (*CuNRT*, *CuNR*, and *CuNIR* encoding putative proteins of 541, 865, and 1,077 residues, respectively) in Ascomycota and Basidiomycota from the subkingdom Dikarya (Materials and Methods) as shown in Figure 2. The phylogenic relationship of the gene cluster in the fungi is similar to the organismal phylogenic position except for the Ascomycete *Trichoderma reesei*, which indeed suggests horizontal gene transfer [32]. Because the three genes of the nitrate/nitrite assimilation pathway of *C. utilis* were strongly suggested to be clustered in Saccharomycetales, we presume that they mediate this assimilation activity in *C. utilis* as well as in *P. angusta*. Although we have yet to perform knock-out studies to confirm this, deletion study of nitrate/nitrite transporters in *P. angusta* was performed and the strain was found not to grow in nitrate media [33]. Comparison of the nitrate/nitrite transporters, nitrate reductases, and nitrite reductases from *C. utilis* and *P. angusta* revealed sequence identifies of 48% (251/522), 58% (492/840) and 61% (637/1043), respectively. On the other hand, the sequential order of the nitrate and nitrite reductase genes in *C. utilis* differs from that in *P. angusta* [33] and the nitrate/nitrite transporter of *C. utilis* is transcribed in the opposite direction relative to that in *P. angusta* [33]. We next constructed a phylogenic tree including *P. angusta* was constructed using 18 single copy orthologous genes in 12 sequenced yeast genomes and *P. angusta* was found to be a part of distinct clade from *C. utilis* (Figure S6).

We next examined the transcription of the three genes of the nitrate/nitrite assimilation pathway based on the RNA-seq data, which was subjected to upper-quartile (uq) normalization [29] (Materials and Methods). The expression of each of the genes, annotated as *CuNRT*, *CuNR*, and *CuNIR*, was greater during log-phase (uq = 0.485, 1.270, and 4.300) than during stationary-phase (uq = 0.059, 0.020, and 0.216), respectively. The expression of *CuNR*, and *CuNIR* was particularly high during the log-phase grown in the nutrient rich media.

### Hexose transporters in *C. utilis*

**Figure 3:**
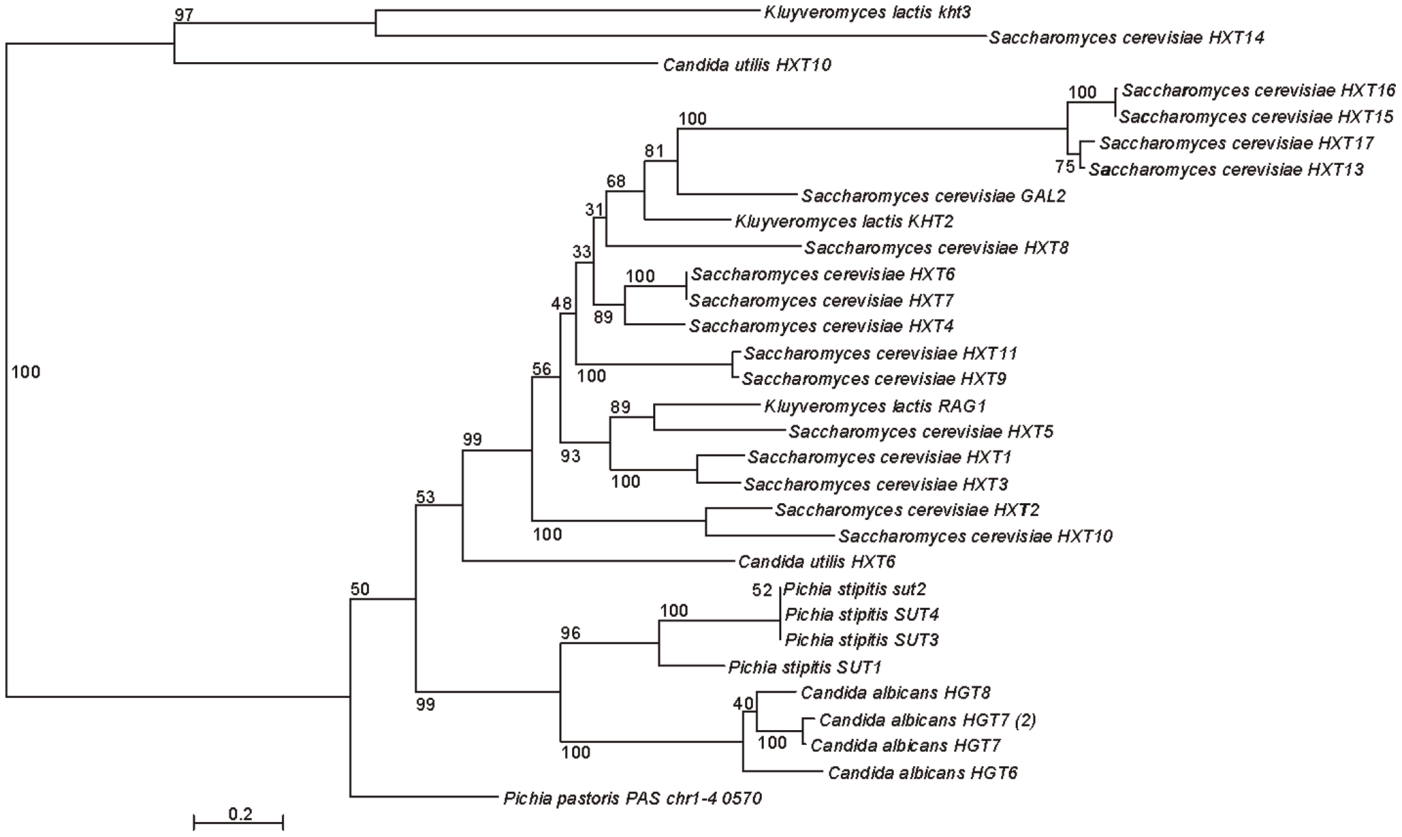
Phylogenetic tree of 31 hexose transporters by the ML method. These were selected from 114 transporters in *C. utilis*, *S. cerevisiae*, *S. pombe*, *P. stipitis*, *A. gossypii*, *K. lactis*, *C. albicans*, *C. glabrata*, *D. hansenii*, and *P. pastoris*.

A phylogenic tree was constructed based on 114 transporters in *C. utilis*, *S. cerevisiae*, *S. pombe*, *P. stipitis*, *A. gossypii*, *K. lactis*, *C. albicans*, *C. glabrata*, *D. hansenii*, and *P. pastoris*. Base on this tree, thirty-one proteins were assumed to be hexose transporters. Next, the phylogenic tree was reconstructed using the 31 transporters and is shown in Figure 3. In contrast to *S. cerevisiae*, which possesses 20 isogenes encoding low- and high-affinity hexose transporters, only two putative hexose transporters are present in the *C. utilis* genome (cut01g0001347 and cut01g0020536). RNA-Seq analysis revealed that cut01g0001347, annotated as *CuHXT6*, was more highly expressed than cut01g0020536 (*CuHXT10*) (Table 3), suggesting that *CuHXT6* functions as a pivotal hexose transporter under both high and low D-glucose concentrations.

**Table 3:** Transcription results of 2 putative hexose transporters of *C. utilis*.

		Normalized RNA-Seq data (upper-quartile)	
Gene ID	Symbol	Log-phase	Stationary-phase
cut01g0001347	CuHxt6	52.84	39.93
cut01g0020536	CuHxt10	0.17	0.16

### Annotated genes involved in ethanol production and assimilation in *C. utilis*

Analysis of the genome sequence revealed that *C. utilis* has six and seven orthologous genes encoding alcohol dehydrogenase (ADH) and aldehyde dehydrogenase (ALD), respectively. We next performed gene expression analysis by RNA-Seq in order to examine which ADH and ALD genes were transcribed (Table 4). the ethanol concentrations and the transcription of ADH genes (Table 4), we presume that cut01g0000110 [named *CuADH1-F* (“F” stands for “forward.”)] functions in the reduction of acetaldehyde to ethanol because the gene was strongly expressed during the log-phase. In contrast, cut01g0050119 (*CuADH2*) and cut01g0021005 (*CuADH3*), which were both more highly expressed during the stationary-phase than during the log-phase, probably play a role in the conversion of ethanol to acetaldehyde. Based on the level of their expression during the log-phase, we presume that the products of these two genes act to assimilate ethanol even in the presence of D-glucose. Furthermore, the product of the ALD gene (*CuALD6*), which functions downstream of ethanol assimilation (the conversion of ethanol to acetaldehyde) through CuAdh2/3 was also highly expressed during both phases (Table 4). We also predicted the cellular localization of these genes using WoLF PSORT [34] as shown in Table 4. Ethanol assimilation in both the cytoplasm and mitochondria is thought to occur during both phases of growth in *C. utilis*, and is believed to contribute to the alleviation of the D-glucose overflow metabolism that is characteristic of Crabtree effect-negative yeast such as *C. utilis*.

**Figure 4:**
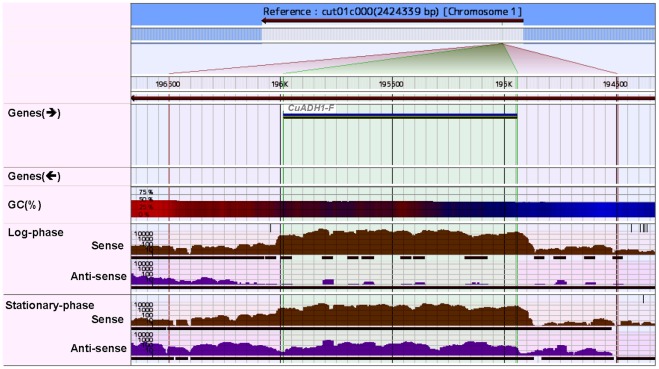
Antisense transcripts of *C. utilis ADH1* (cut01g0000110) differentially expressed between log- and stationary-phases. Expressions at the *ADH1* locus was visualized using the Genaris integrated Next-Generation Sequencing Data Analysis Platform (GiNeS) (Genaris, Inc., Kanagawa, Japan).

In our genome sequencing, *C. utilis* was found to lack the conserved components required for RNA interference (RNAi), including Dicer-like RNase and Argonaute which were found in several budding yeast, such as *Saccharomyces castellii* and *Candida albicans* [35], [36]. However, some budding yeasts including *S. cerevisiae*, have been reported to have genes that are regulated by non-coding RNAs [37]. In the present study, antisense transcripts in *C. utilis* were analyzed using the RNA-seq data, which revealed a novel antisense transcript complementary to the entire *CuADH1-F* region, shown in Table 4 and Figure 4. Although the expression of *CuADH1-F* during the stationary-phase was one-sixth lower than during log-phase, the expression of the antisense transcript [*CuADH1-R* (“R” stands for “reverse.”)] during the stationary-phase was 93.5-fold greater than during the log-phase. In addition, the number of tags aligned at *CuADH1-R* (226,551) was 1.6-fold greater than those at *CuADH1-F* (145,562) during the stationary-phase. The inverse correlation between the expression of these two overlapping transcripts strongly suggests a possible mechanism whereby the expression of the antisense transcript acts to repress the expression of *CuADH1-F*.

## Discussion

In the present study, we have conducted genome sequencing and whole genome transcriptome analysis of the *C. utilis* NBRC0988 strain. Although the phylogenic position has been already reported based on analysis of ribosomal DNA [14]–[16], we constructed a phylogenic tree, to clarify this phylogenic position using whole genome sequence data. We conclude that *C. utilis* represents a distinct clade among the 13 yeasts analyzed and that it diverged before the *Saccharomyces*/*Kluyveromyces* clade and long before the formation of the CUG clade.

**Table 4:** *C. utilis* ADH and ALD genes, and transcription results.

			Normalized RNA-Seq data (upper-quartile)			
			Log-phase		Stationary-phase	
Gene ID	Symbol	Site*	Sense	Antisense	Sense	Antisense
cut01g0000110	CuAdh1-F	Cyto.	134.37	0.88	23.92	81.83
cut01g0050119	CuAdh2	Mito.	8.56	0.31	26.35	5.66
cut01g0021005	CuAdh3	Cyto.	12.63	0.94	68.16	5.41
cut01g0020374	CuAld6-c	Cyto.	7.93	0	8.08	0
cut01g0030970	CuAld5	Mito.	2.40	0.52	0.22	0.071
cut01g0050658	CuAld6-m	Mito.	9.10	0.52	62.11	0.33

* Protein localization site was predicted by WoLF PSORT [34].

Cyto.: Cytoplasm, Mito.: Mitochondrion.

We obtained a new finding about nitrate assimilation related genes in *C. utilis* and its phylogenic position among other microbes. As a noteworthy characteristic, *C. utilis* has been reported to assimilate nitrate and then to convert nitrite to ammonia as a nitrogen source [38]. This metabolic pathway is industrially useful since nitrate is a naturally occurring mineral source of nitrogen that can be found in the form of potassium nitrate. Thus, *C. utilis* can survive some poor conditions where other yeasts including *S. cerevisiae* can not survive. The amino acid sequences of *C. utilis* nitrate reductase, nitrite reductase, and nitrate/nitrite transporter are all homologous to the correspding enzymes in *P. angusta* and the corresponding genes are clustered in Saccharomycetales (Figure 2). However, the putative order of the nitrate and nitrite reductase genes differs between the two yeasts, and the nitrate/nitrite transporter gene in *C. utilis* is located in the opposite direction compared to that in *P. angusata* [33]. In addition, *C. utilis* and *P. angusta* are classified within distinct clades of the phylogenetic tree (Figure S6). In conclusion, *C. utilis* has maintained the capacity for nitrate assimilation and it is likely that the loss of nitrate assimilation genes in other yeasts analyzed clustered in Saccharomycetales occurred in both the clades containing *C. utilis* and *P. angusata*, respectively.

*C. utilis* is Crabtree effect-negative which is partially derived from limitation of sugar uptake to avoid overflow in metabolism [39]. Hence, we focused on hexose transporters in *C. utilis*. As shown in Figure 3, the phylogenic analysis revealed that *C. utilis* had fewer hexose transporters than *S. cerevisiae*. In addition, the gene cut01g0001347, annotated as *CuHxt6*, is assumed to be a pivotal hexose transporter at both high and low concentrations of D-glucose. The protein encoded by CuHxt6 exhibits high sequence similarity (more than 63% identity) to the Crabtree effect-negative yeast *K. lactis* hexose transporter 2 (*Kht2*), which has an intermediate affinity for D-glucose (K_m_ of 3.7 mM) [40]. Although *K. lactis Hgt1* is a high-affinity D-glucose transporter (K_m_ of 1 mM) [41] and homologous (27.8% identity) to *C*. *utilis Hgt1* (cut01g0021027), it is poorly transcribed during both phases (uq = 0.088 at log-phase and uq = 0.0066 at stationary-phase). On the other hand, no low-affinity hexose transporter was predicted in the genome of *C. utilis*. These data suggest that *C*. *utilis* limits its uptake of D-glucose, and this is consistent with previous reports indicating that *C. utilis* only uses a high affinity D-glucose uptake system [39] and that Crabtree-effect negative yeasts generally exhibit K_m_ values for D-glucose in the micromolar range [42].

The *C. utilis* transcriptome during the log- and stationary-phases included more than four thousand transcripts within either intergenic or antisense regions (Table 2), which had not been predicted by any *in silico* approach. There have been previous reports of antisense transcriptional regulation in yeasts [43]–[45] which may play a different role in acting post-transcriptionally by RNAi mechanisms. In *S. cerevisiae*, zinc deficiency has been shown to induce the expression of the transcription factor Zap*1*, which in turn induces expression of intergenic RNA and repression of *ADH1* gene expression [46]. However, we did not identify any *Zap1* orthologs in *C. utilis*. Since the antisense transcript *CuADH1-R* is expressed during the stationary-phase but not the log-phase, we propose an alternative hypothesis that this transcript is involved in repression of ethanol production during the stationary-phase. Our results appear to differ from a previous report [46], because we believe that *CuADH1-F* may be repressed by its antisense transcript during the stationary-phase. Suppression mechanism of antisense transcripts has been reported to be enforced by two distinct mechanisms in *S. cerevisiae*: Rpd3S-mediated deacetylation that prevents antisense initiation [47], and Nrd1–Nab3–Sen1 complex-mediated termination [48]. Since *C*. *utilis* lacks the conserved components required for RNA interference (RNAi), including Dicer-like RNase and Argonaute as is the same with *S. cerevisiae*, we paid attention to the four homologs in *C. utilis* genome to reveal that all the four were actually transcribed. We detected the RNA-Seq tags in *CuRPD3*: (11,002 at log-phase and 12,059 at stationary-phase), *CuNRD1*: (20,513 and 44,159), *CuNAB3* (: 17,071 and 35,917), and *CuSEN1*: (10,541 and 13,082). Herein, there might be a possibility that the expression of *CuADH1-R* might be controlled by either of the two mechanisms in *C. utilis*.

RNA-Seq is not limited to detecting transcripts that correspond to existing exon sequences, but also reveals the precise genome locations of transcripts corresponding to non-coding regions by comparison with genome reference information. A second advantage of RNA-Seq relative to microarray analysis is that it gives rise to very low background signals because the DNA sequences can be unambiguously mapped to unique regions of the genome. In addition, it can detect a large dynamic range of expression [49]. These advantages have led here to the identification of genes that play central roles in hexose transport and of a novel antisense transcript (*CuADH-R*). We believe that our RNA-Seq data has provided tremendous insight into and should help inform the design of follow-up experiments that aim to clone novel genes and useful promoters, quantitative trait locus (eQTL) mapping, and so on.

The wealth of information provided by complete genome sequence and transcriptome analysis will enable us to develop more sophisticated systems for the production of heterologous proteins and novel compounds. The genome data of *C. utilis* reported in the present study are now publicly available, and we expect this information to generate novel findings and developments in the food and pharmaceutical industries.

## Materials and Methods

### DNA preparation

*C. utilis* NBRC0988 (ATCC 9950, CBS 5609) was obtained from the National Institute of Technology and Evaluation (NITE) Biological Resource Center. This particular yeast strain was cultured at 30°C in YPD medium (1% yeast extract, 2% peptone, and 2% dextrose). Genomic DNA (>300 μg) was prepared according to a published protocol (Molecular Cloning 3^rd^ edition). A *C. utilis* DNA library for sequencing was prepared by random cleavage of its DNA using a Hydroshear Device (GeneMachines, San Carlos, CA). Plasmid and fosmid DNA libraries comprised fragments of approximately 3 to 4-kb and 40-kb, respectively.

### Genome sequencing and assembly

#### Shotgun sequencing (Sanger)

DNA templates were sequenced in a 384-well format, with the forward and reverse reactions (paired ends) being performed on the same plate to maximize the paired end pass rate. BigDye^TM^ Terminator version 3.1 (Applied Biosystems, Foster City, CA) reactions were used in preparation for sequencing on the ABI PRISM 3730xl. Thermal cycling was performed using 384-well Thermocyclers (Applied Biosystems). Sequencing reactions were purified using the CleanSeq dye-terminator removal kit (Agencourt, Danvers, MA). A passing read was defined as an average high quality PHRED score of 20 or greater for at least 100 bases, producing 57.9 Mb from plasmid and 18.5 Mb from fosmid libraries (read length: 500–600 bp).

#### Pyrosequencing and assembly using a hybrid approach

We also used the GS-FLX (Roche) to produce 211.7 Mb and generate 1,017-K pair-end reads with 250 bp (averaged depth 20), to enable assembly with the Newbler assembler version 1.1.03.24 that is a part of the software package distributed with the sequencing machines. Subsequently, we obtained 975 supercontigs (total supercontig length: 14.6 Mb) through both the GS-FLX and the Sanger sequencing data with the Arachne assembler version 2.0 [50], respectively.

#### Codon usage

Nucleotide sequences of the predicted 8,646 ORFs in *C. utilis* and the 5,865 ORFs in *S. cerevisiae* S288C strain were analyzed with ANACONDA 2.0 [51] to calculate codon usage.

### Genome sequence annotation

Protein coding genes were predicted using GlimmerM [19] and GeneLook [20] from 975 supercontigs (Figure S1). The *Saccharomyces* genome database (SGD) (www.yeastgenome.org) was used as training data for GlimmerM, resulting in the prediction of 10,560 genes. Meanwhile, 8,927 genes were predicted using the *ab initio* gene finder, GeneLook. Among the genes designated by the two methods, we first selected those that were assumed to contain less than 10 consecutive nucleotide gaps and to comprise 60 or more amino acids. Among the latter, we subsequently selected common 4,536 genes in terms of strand direction, transcriptional initiation, and termination sites. In addition, another 598 and 1,757 genes were predicted individually (Figure S1). Those genes predicted by one method that overlapped partially with genes predicted by another method, but with less annotation accuracy (Figure S2) were not analyzed further. In sum, a total of 8,646 protein-coding genes were annotated using the SGD (6,717 ORFs), the *Candida* Genome Database (CGD) (www.candidagenome.org) (6,107 ORFs), and SwissProt release 55.6 (788,247 proteins). These were subsequently classified into four categories (conserved, slightly conserved, hypothetical, and predicted protein) according to their annotation accuracy (Figure S2).

### Phylogenic tree construction

A phylogenic tree was constructed from the alignment of 32 proteins in Candida utilis NBRC0988, Saccharomyces cerevisiae S288C [23], Schizosaccharomyces pombe 972h- [24], Pichia stipitis CBS6054 [25], Yarrowia lipolytica CLIB122 [26], Ashbya gossypii ATCC10895 [27], Kluyveromyces lactis NRRL Y-1140 [26], Candida albicans SC5314 [28], Candida glabrata CBS138 [26], Debaryomyces hansenii CBS767 [26], Pichia pastoris GS115 [30], Kluyveromyces thermotolerans CBS6340 [52], Saccharomyces kluyveri CBS3082 [52], and Zygosaccharomyces rouxii CBS732 [52], the whole genomes of which have been completely sequenced (Figure 1). Thirty-two proteins were selected from universal single-copy orthologous genes exhibiting synteny in Yeast Gene Order Browser YGOB [53]–[55]. Also, eighteen proteins were selected in 12 yeast genomes including P. angusta under the same condition (Figure S6). The amino acid sequences of members of individual orthologous gene families were aligned using the MUSCLE 3.6 program [56], [57] and these sequences were concatenated together so as to yield an alignment of 19,371 amino acid including gaps. A phylogenic tree (Figure 1 and Figure S6) was constructed on the concatenated sequence by the maximum likelihood (ML) method using RAxML 7.2.8 with a JTT + Γ (gamma) model [58]. Gaps were considered to be complete deletions, and 1,000 bootstrap replicates were performed resulting in a fully resolved tree with all but one node having bootstrap values of 100. Similarly, multiple sequence alignments of the sequences of the genes involved in nitrate assimilation and the 31 hexose transporters were performed using the MUSCLE program [56], [57], and a phylogenic tree was also constructed using the ML method using RAxML 7.2.8 with a JTT + Γ (gamma) model [58] (Figure 2 and Figure 3). In order to identify orthologous genes involved in nitrate assimilation in Ascomycota and Basidiomycota, we performed a BLASTP search comparing each of the three genes involved in this pathway in C. utilis against the NCBI protein database (e-values were each less than 1e-40) and constructed a phylogenic tree (Figure 2).

### Comparative analysis of protein families

In order to classify genes encoding proteins falling within specific families (orthologous and paralogous genes), we compared the amino acid sequence similarity of all proteins among 10 yeasts (*C. utilis*, *S. cerevisiae*, *S. pombe*, *P. stipitis*, *Y*. *lipolytica*, *A. gossypii*, *K. lactis*, *C. albicans*, *C. glabrata*, and *D. hansenii*) under two conditions: All-against-all BLASTP with an e-value of 1e-20 and a cover rate calculated by a total length of high-scoring segment pairs of more than 50%. We subsequently formed a triangle cluster when an orthologous relationship (*e.g.*, protein A to protein C, protein B to protein C) was detected. In those cases where two triangle clusters shared 2 ORFs, we combined the two clusters and this procedure was continued iteratively so as to construct the final protein families [59].

### SOLiD sequencing and mapping

To characterize the *C. utillis* transcriptome, total RNA was extracted from the NBRC0988 strain under aerobic conditions at 25°C in YPD medium (1% yeast extract, 2% peptone, and 5% D-glucose) during both log-phase with remaining D-glucose (10.5 h) and during stationary-phase when the D-glucose was depleted (24 h) using the RNeasy Mini Kit (QIAGEN GmbH., Hilden, Germany). Poly (A) RNA was isolated from the total RNA by using Poly (A) Purist^TM^ mRNA Purification Kit (Ambion). The poly (A)-selected RNA was fragmented by incubation with RNase III (Ambion), and 100 to 200-bp fragments were separated by gel electrophoresis. According to the standard protocol described in the SOLiD Library Preparation Guide, cDNA was amplified onto beads and was subjected to emulsion PCR. Each library was sequenced using SOLiD^TM^ 3 to generate 50-bp long reads according to the supplied protocol.

Sequence data were mapped and aligned to the reference genome using BioScope^TM^ software 1.0. The obtained sequences comprised 50-bp reads and they were divided into the former 25-bp (5′) and latter 25-bp (3′) and were required to map uniquely to the *C. utilis* genome, allowing up to two mismatches at the former 25-bp and no insertions or deletions. There are three parallel mappings: mapping to filter sequences [filtering out ribosomal RNA (about 1.5% and 2.1 % in the mapped tags in log- and stationary-phase samples, respectively)], mapping to the reference genome (exon sequences defined by the genome annotation), and mapping to splice junctions (defined by the genome annotation). The number of tags mapping to unique regions of the genome were used as an estimation of quantitative expression of the particular genes.

### Determination of normalized gene expression by RNA-Seq

The expression levels of genes determined by RNA-Seq were subjected to upper-quartile (uq) normalization [29] for comparison of expression between log- and stationary-phases. The upper quartile (75th percentile) was computed for each sample ( =  each column), after excluding genes with zero tags across all the columns. The normalized count equals the raw count divided by the upper quartile. We also applied this normalization method to the analysis of antisense transcript expression separately from the aligned tags at ORFs.

### Data access

The *C. utilis* genomic sequence has been deposited in the DDBJ/EMBL/GenBank under accession numbers BAEL01000001-BAEL01001163 and DG000065-DG000077. RNA-Seq data have been deposited in the DDBJ Sequence Read Archive (DRA) under accession number DRA000498.

## Supporting Information

Figure S1 **Summary of integrated gene prediction platform.** Annotation accuracy is indicated in Figure S2.
(TIFF)

Figure S2 **Summary of gene sequence annotation**.
(TIFF)

Figure S3 **Functional comparison of (A)** ***C. utilis*** **and (B)** ***S. cerevisiae*** **according to KOG categories.** The rate of genes categorized to the following 3 KOGs in *C. utillis* is more than 1% compared to that of *S. cerevisiae*: *amino acid transport and metabolism (279, 216), **lipid transport and metabolism (226, 173), and ***secondary metabolites biosynthesis, transport and catabolism (135, 80). The figures in the brackets represent the number of genes of *C. utillis* and *S. cerevisiae*, respectively.
(TIFF)

Figure S4 **(A)** ***C. utilis*** **and (B)** ***S. cerevisiae*** **codon usage**.
(TIFF)

Figure S5 **Venn diagram grouping protein families in** ***C. utilis*** **(A),** ***S. cerevisiae*** **(B), and** ***C. albicans*** **(C).** The 2,397 of the protein families in *C. utilis* belonged to families that were present in either *S. cerevisiae* or *C. albicans*, or in both shown in ABC, ABc, and AbC.” The 4,894 families were found to lack homologous sequences in *S. cerevisiae* and *C. albicans*. Each lower-case character (a, b, and c) represents a group of complementary families in each yeast.
(TIFF)

Figure S6 **Phylogenetic tree of single copy orthologous genes in 12 species.** This tree was built on concatenated sequence of 18 single copy orthologous genes in 12 species by the ML method using RAxML 7.2.8 with a JTT + Γ (gamma) model [58].
(TIFF)

Table S1 **Overview of Pfam domains specific to C. utilis compared to nine other yeasts (** ***S. cerevisiae, S. pombe, P. stipitis, Y. lipolytica, A. gossypii, K. lactis, C. albicans, C. glabrata,*** **and** ***D. hansenii*** **)**.
(PDF)

Table S2 **Annotated proteins of C. utilis without homologous sequences in ten other yeast genomes (** ***S. cerevisiae, S. pombe, P. stipitis, Y. lipolytica, A. gossypii, K. lactis, C. albicans, C. glabrata, D. hansenii, and P. pastoris*** **) detected with a tBLASTN search (e-value 1e-10)**.
^*^ Proteins without any annotations in other yeast genomes.
(PDF)

Table S3 **KEGG pathways in which more than 10% of enzymatic genes were classified into high expression genes (uq>10) at log-phase**.
(PDF)

Table S4 **KEGG pathways in which more than 10% of enzymatic genes were classified into high expression genes (uq>10) at stationary-phase**.
(PDF)

Table S5 **Metabolic pathways defined in the KEGG database in which either** ***S. cerevisiae*** **or** ***C. albicans,*** **or both, lack one or more of the genes in** ***C. utilis*** **within the pathway**.
(PDF)
