# Supplementary figures and images for: Genome and Transcriptome Analysis of the Food-Yeast Candida utilis
Source: PLoS One. 2012 May 18;7(5):e37226. doi: 10.1371/journal.pone.0037226 (PMC3356342; doi:10.1371/journal.pone.0037226)

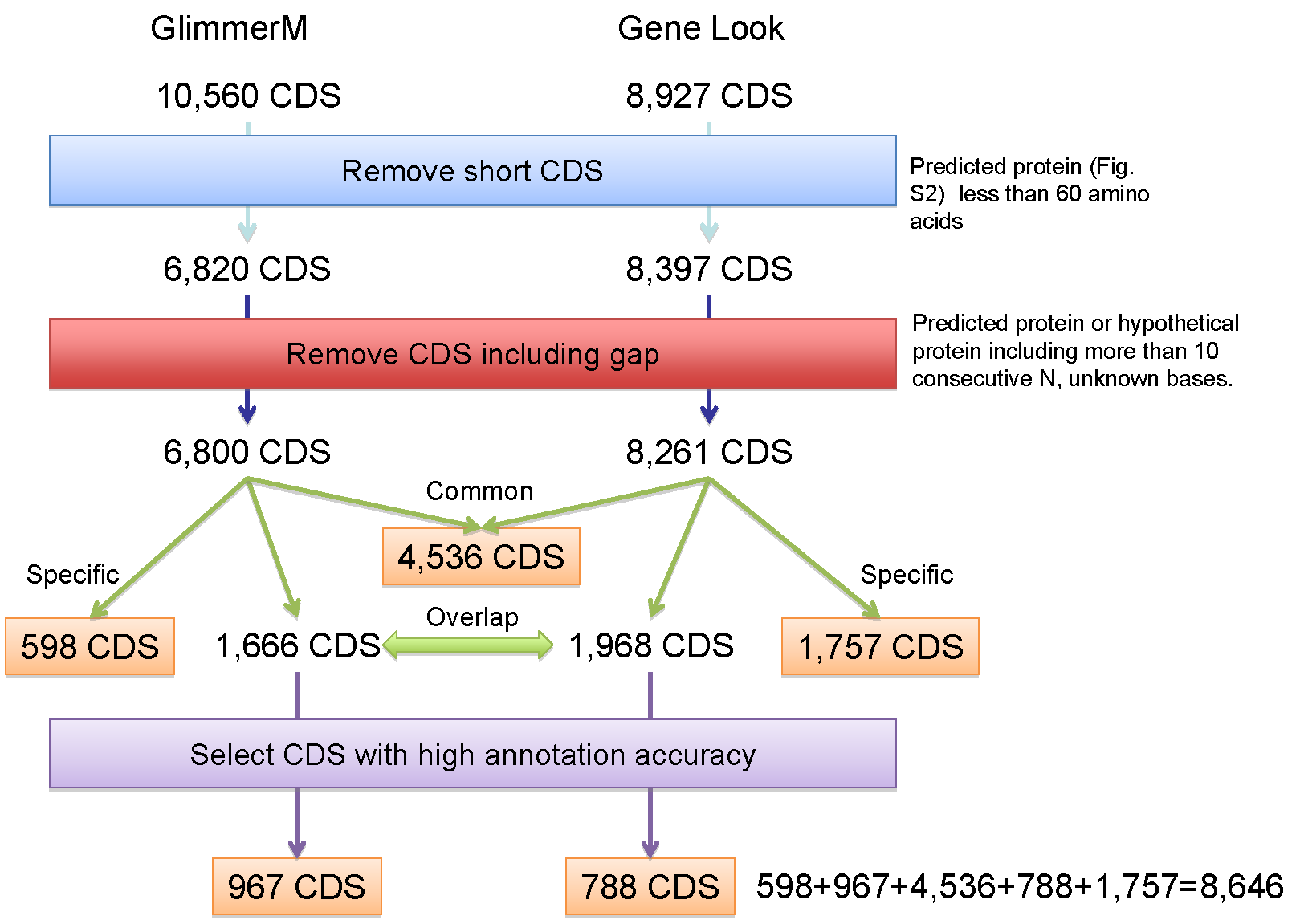

Supplement: Figure S1 — Summary of integrated gene prediction platform. Annotation accuracy is indicated in Figure S2. (TIFF) [file pone.0037226.s001.tiff]

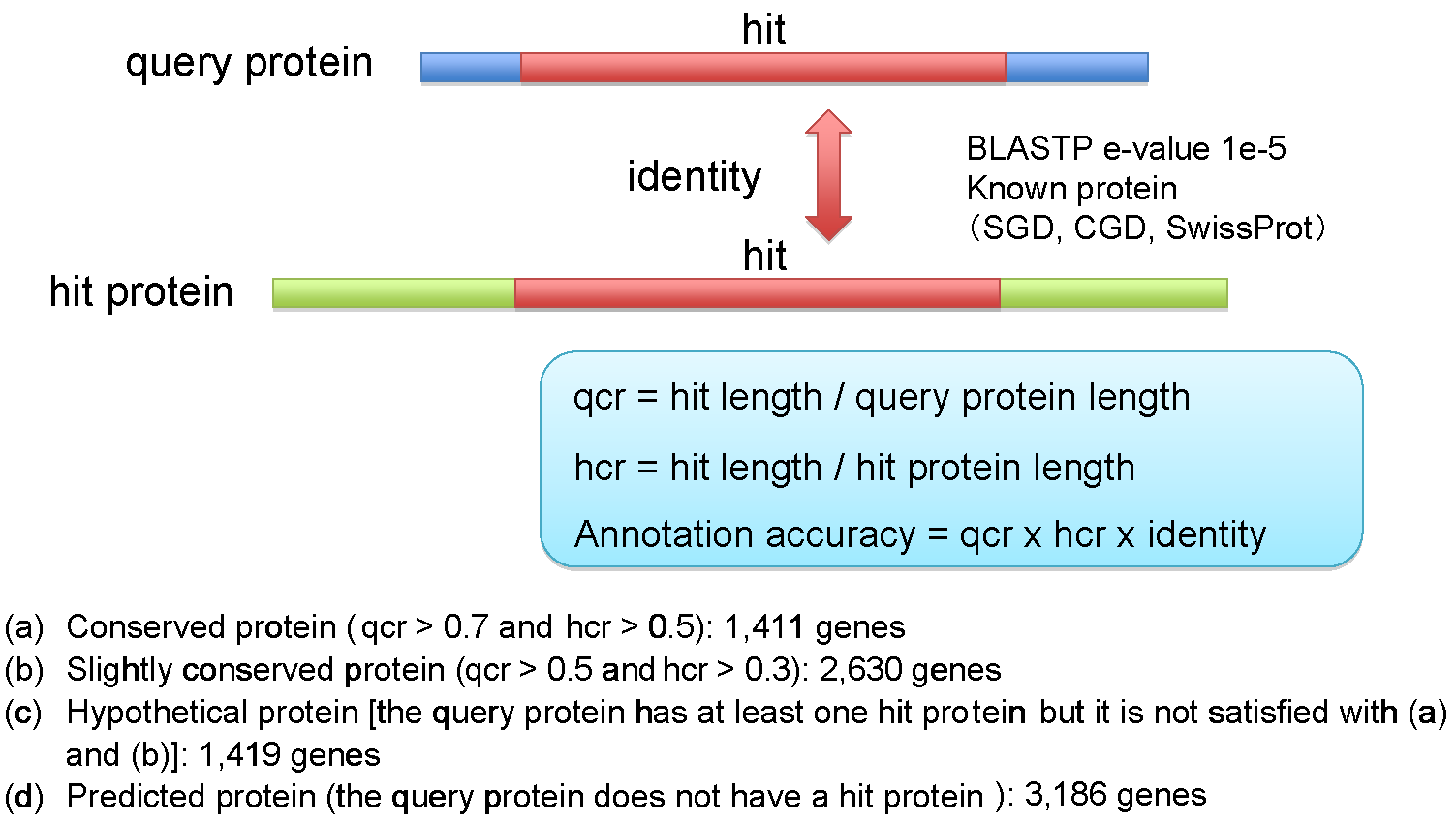

Supplement: Figure S2 — Summary of gene sequence annotation. (TIFF) [file pone.0037226.s002.tiff]

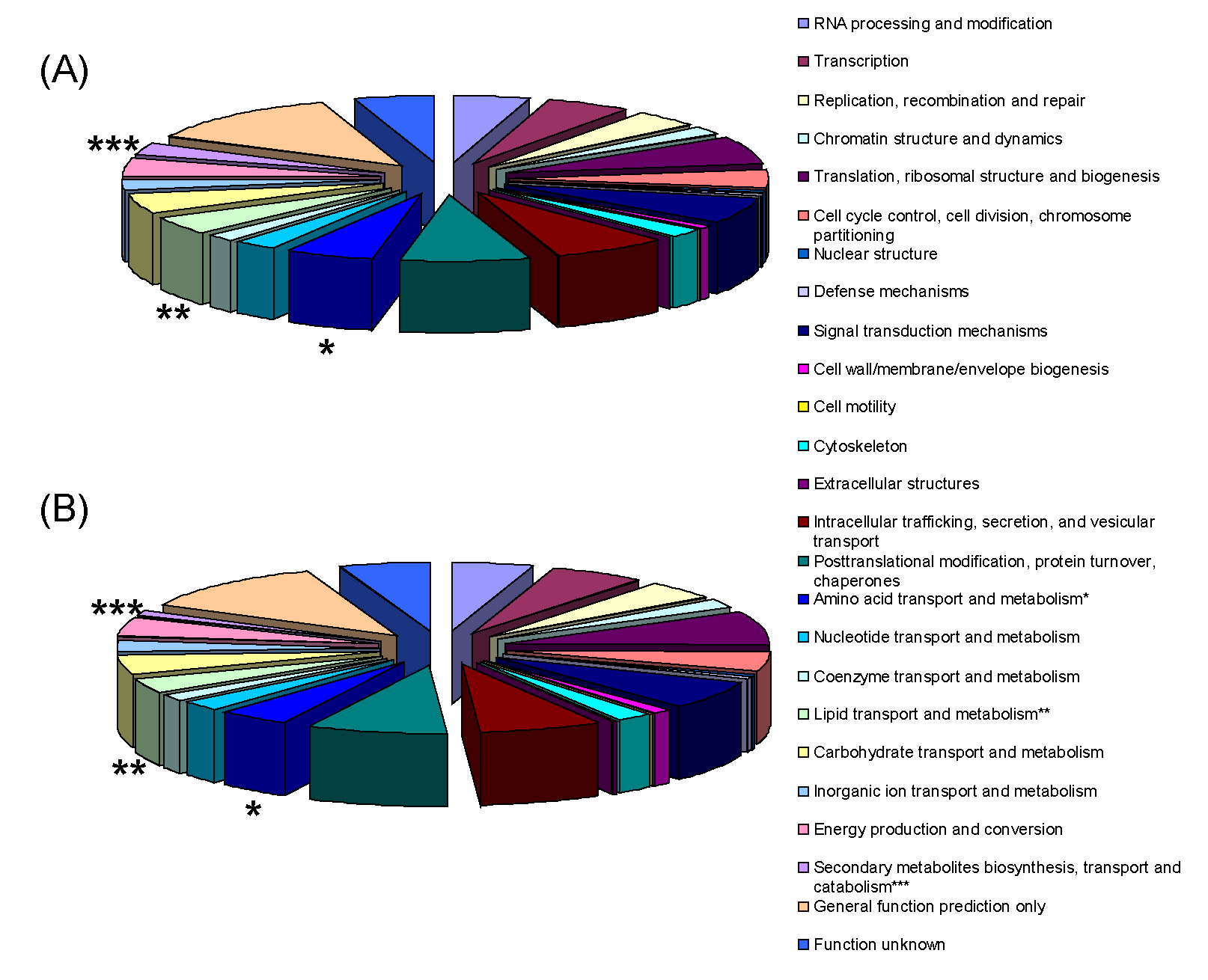

Supplement: Figure S3 — Functional comparison of (A) C. utilis and (B) S. cerevisiae according to KOG categories. The rate of genes categorized to the following 3 KOGs in C. utillis is more than 1% compared to that of S. cerevisiae: *amino acid transport and metabolism (279, 216), **lipid transport and metabolism (226, 173), and ***secondary metabolites biosynthesis, transport and catabolism (135, 80). The figures in the brackets represent the number of genes of C. utillis and S. cerevisiae, respectively. (TIFF) [file pone.0037226.s003.tiff]

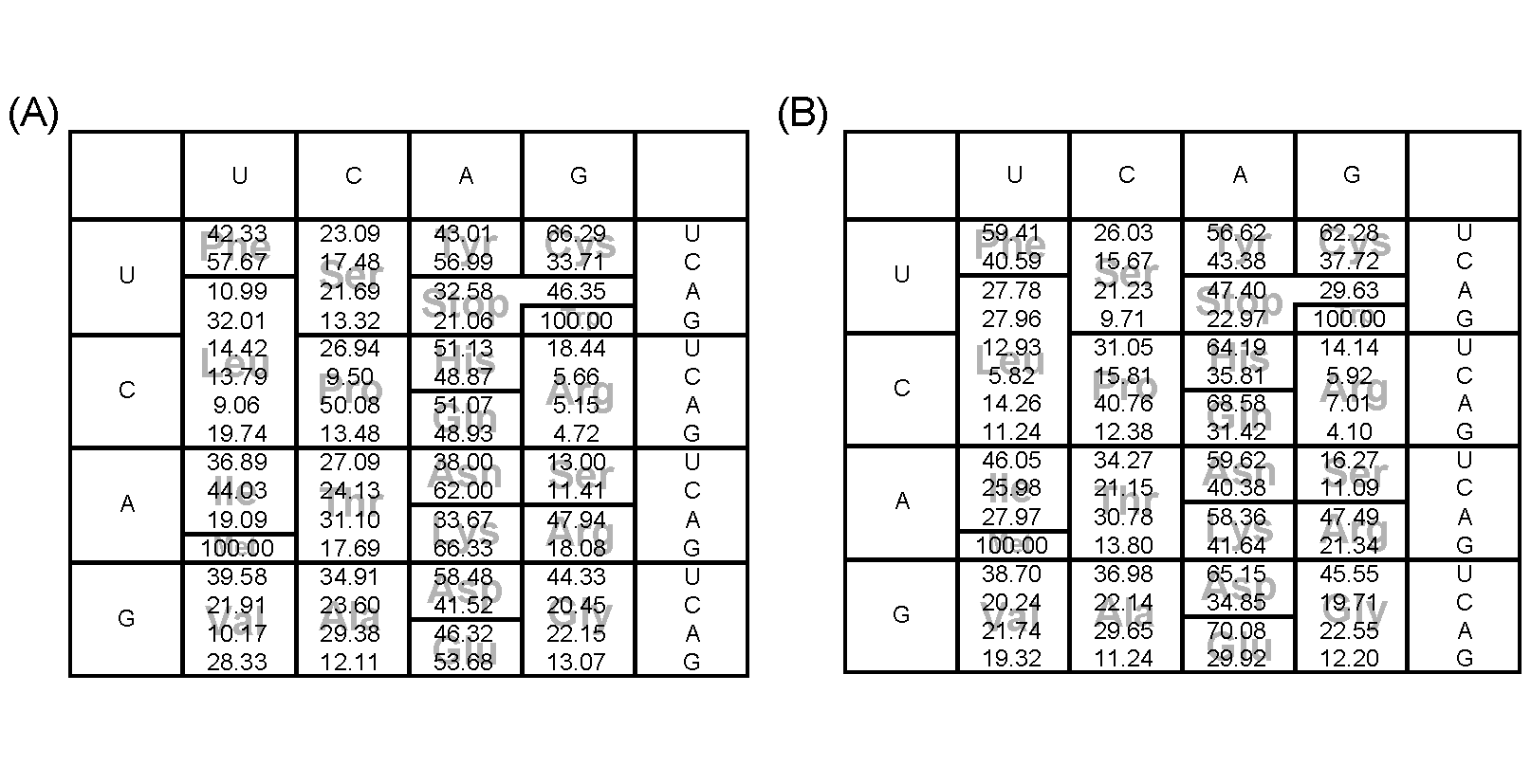

Supplement: Figure S4 — (A) C. utilis and (B) S. cerevisiae codon usage. (TIFF) [file pone.0037226.s004.tiff]

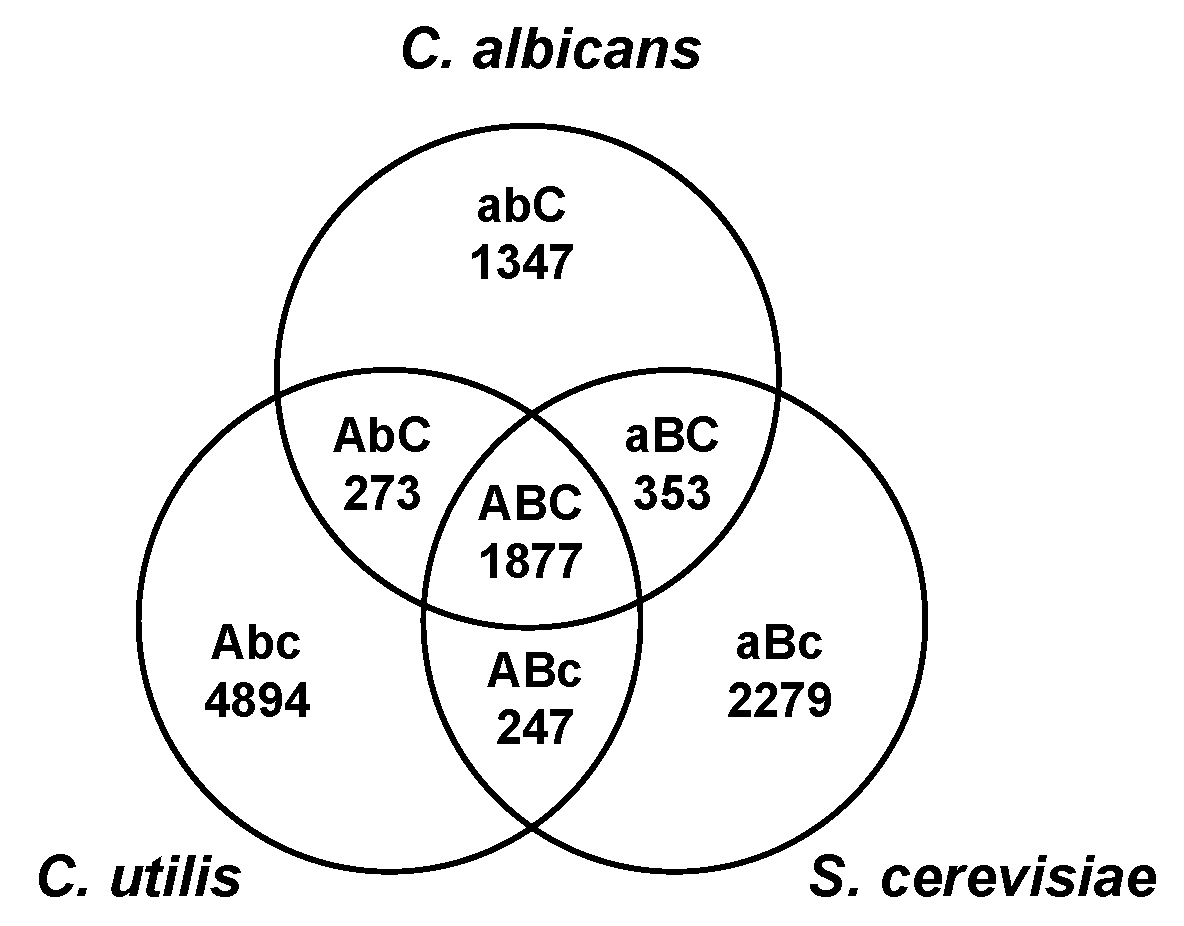

Supplement: Figure S5 — Venn diagram grouping protein families in C. utilis (A), S. cerevisiae (B), and C. albicans (C). The 2,397 of the protein families in C. utilis belonged to families that were present in either S. cerevisiae or C. albicans, or in both shown in ABC, ABc, and AbC.” The 4,894 families were found to lack homologous sequences in S. cerevisiae and C. albicans. Each lower-case character (a, b, and c) represents a group of complementary families in each yeast. (TIFF) [file pone.0037226.s005.tiff]

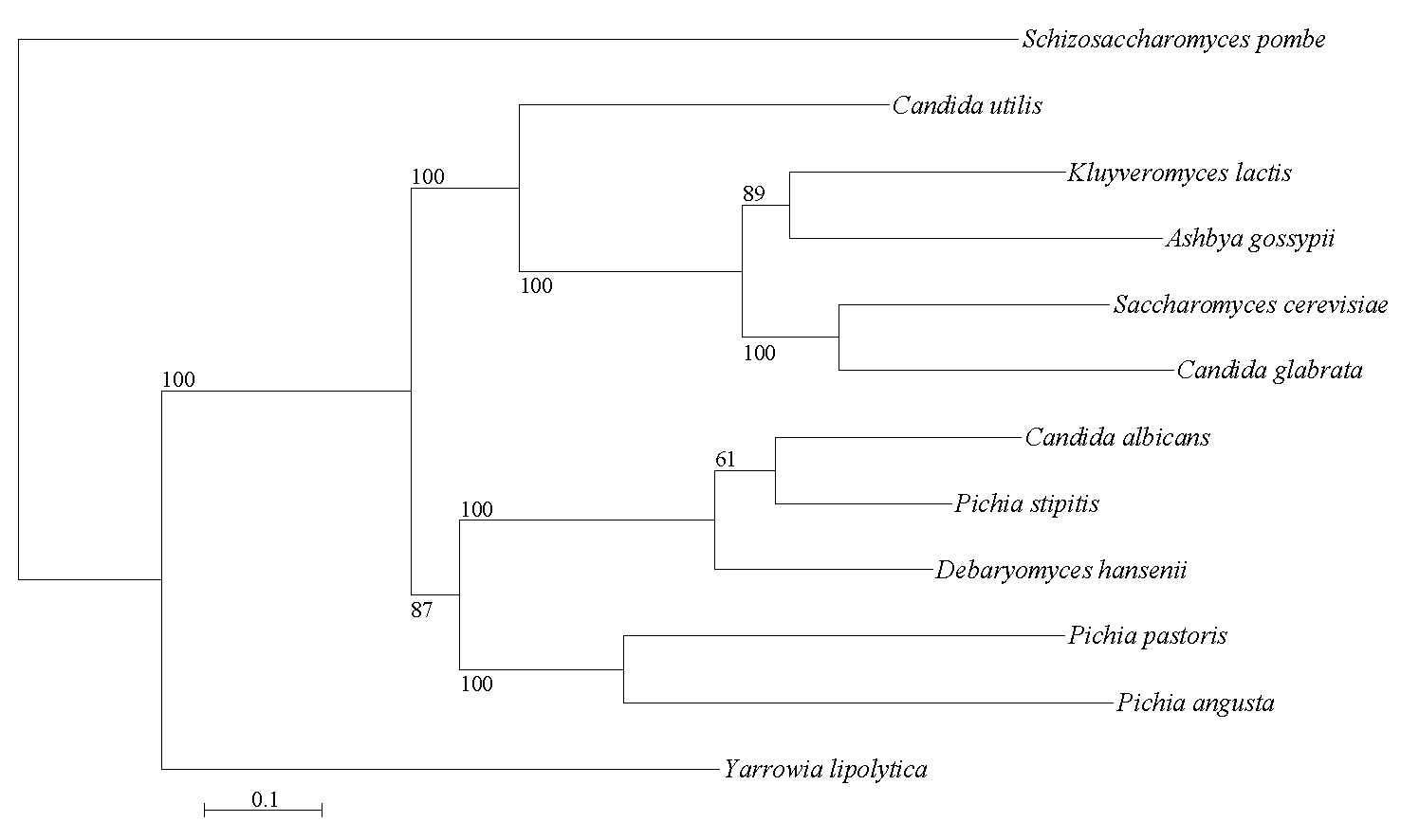

Supplement: Figure S6 — Phylogenetic tree of single copy orthologous genes in 12 species. This tree was built on concatenated sequence of 18 single copy orthologous genes in 12 species by the ML method using RAxML 7.2.8 with a JTT + Γ (gamma) model [58]. (TIFF) [file pone.0037226.s006.tiff]
