# Supplementary material for: Genome and Transcriptome Analysis of the Food-Yeast Candida utilis
Source: PLoS One. 2012 May 18;7(5):e37226. doi: 10.1371/journal.pone.0037226 (PMC3356342; doi:10.1371/journal.pone.0037226)
Supplement: Table S1 — Overview of Pfam domains specific to C. utilis compared to nine other yeasts ( S. cerevisiae, S. pombe, P. stipitis, Y. lipolytica, A. gossypii, K. lactis, C. albicans, C. glabrata, and D. hansenii ). (PDF) [file pone.0037226.s007.pdf]

Table S1. Overview of Pfam domains specific to *C. utilis* compared to nine other yeasts (*S. cerevisiae*, *S. pombe*, *P. stipitis*, *Y. lipolytica*, *A. gossypii*, *K. lactis*, *C. albicans*, *C. glabrata*, and *D. hansenii*).

PfamID	Pfam description	Gene
PF09248	Domain of unknown function (DUF1965)	1
PF02162	XYPPX repeat (two copies)	2
PF05970	PIF1 helicase	1
PF03107	C1 domain	1
PF03476	MOSC N-terminal beta barrel domain	1
PF03473	MOSC domain	1
PF05676	NADH-ubiquinone oxidoreductase B18 subunit (NDUFB7)	1
PF00089	Trypsin	1
PF02391	MoaE protein	1
PF07156	Prenylcysteine lyase	1
PF09423	PhoD-like phosphatase	1
PF01967	MoaC family	1
PF06463	Molybdenum Cofactor Synthesis C	1
PF00174	Oxidoreductase molybdopterin binding domain	1
PF03404	Mo-co oxidoreductase dimerisation domain	1
PF08914	Rap1 Myb domain	1
PF02755	RPEL repeat	1
PF08445	FR47-like protein	1
PF03453	MoeA N-terminal region (domain I and II)	1
PF03454	MoeA C-terminal region (domain IV)	1
PF05336	Domain of unknown function (DUF718)	1
PF07016	Cysteine-rich acidic integral membrane protein precursor	1
PF05658	Hep_Hag	1
