# Supplementary material for: Genome and Transcriptome Analysis of the Food-Yeast Candida utilis
Source: PLoS One. 2012 May 18;7(5):e37226. doi: 10.1371/journal.pone.0037226 (PMC3356342; doi:10.1371/journal.pone.0037226)
Supplement: Table S2 — Annotated proteins of C. utilis without homologous sequences in ten other yeast genomes ( S. cerevisiae, S. pombe, P. stipitis, Y. lipolytica, A. gossypii, K. lactis, C. albicans, C. glabrata, D. hansenii, and P. pastoris ) detected with a tBLASTN search (e-value 1e-10). * Proteins without any annotations in other yeast genomes. (PDF) [file pone.0037226.s008.pdf]

Table S2. Annotated proteins of *C. utilis* without homologous sequences in ten other yeast genomes (*S. cerevisiae*, *S. pombe*, *P. stipitis*, *Y. lipolytica*, *A. gossypii*, *K. lactis*, *C. albicans*, *C. glabrata*, *D. hansenii*, and *P. pastoris*) detected with a tBLASTN search (e-value 1e-10).

Gene ID	Symbol	Annotation
cut01g0080002	NuoN	NADH:ubiquinone oxidoreductase subunit 2 (chain N).
cut01g0080007	VAR1	Mitochondrial ribosomal protein VAR1.
cut01g0080011	ND5	NADH-ubiquinone oxidoreductase chain 5 (EC 1.6.5.3).
cut01g0080012	ND4L	NADH-ubiquinone oxidoreductase chain 4L (EC 1.6.5.3).
cut01g0080013	ND1	NADH-ubiquinone oxidoreductase chain 1 (EC 1.6.5.3).
cut01g1480001	2-NPD	2-nitropropane dioxygenase (EC 1.13.11.32).
cut01g0000019	SNM1	Ribonuclease MRP protein subunit.
cut01g0000031	PEX22	Peroxisome assembly protein 22 (Peroxin-22).
cut01g0000352	eif3h	Translation initiation factor 3, subunit h.
cut01g0000458	no symbol	AAA ATPase containing von Willebrand factor type A (vWA) domain.
cut01g0001540	no symbol	DEAD box containing helicases.
cut01g0010286	no symbol	Conserved WD40 repeat-containing protein.
cut01g0010764	no symbol	Adaptor complexes medium subunit family.
cut01g0010818	no symbol	Uncharacterized protein YLR363W-A.
*cut01g0010836	Pcyox1	Probable prenylcysteine oxidase precursor (EC 1.8.3.5).
cut01g0010850	cwf18	Cell cycle control protein cwf18.
cut01g0010986	SPT2	Protein SPT2 (Negative regulator of Ty transcription).
*cut01g0011116	PhoD	Phosphodiesterase/alkaline phosphatase D.
*cut01g0011260	crnA	Nitrate/nitrite transporter.
cut01g0020370	no symbol	Predicted membrane protein.
cut01g0020795	YAP3	AP-1-like transcription factor YAP3.
cut01g0020940	no symbol	Uncharacterized protein YMR184W.
cut01g0030299	MRPL13	54S ribosomal protein L13, mitochondrial precursor (YmL13).
cut01g0030328	TVP23	Golgi apparatus membrane protein TVP23.
cut01g0030339	SMD3	Small nuclear ribonucleoprotein Sm D3.
cut01g0030493	HSP150	150 kDa heat shock glycoprotein precursor.
cut01g0030838	no symbol	Myosin class II heavy chain.
cut01g0030996	YPI1	Type 1 phosphatases regulator YPI1.
cut01g0031169	TIM22	Mitochondrial import inner membrane translocase subunit TIM22.
cut01g0040151	CWC15	Pre-mRNA-splicing factor CWC15.
cut01g0040214	SNU71	U1 small nuclear ribonucleoprotein component SNU71.
cut01g0040387	CSN4	COP9 signalosome, subunit CSN4
cut01g0050534	Gst	Glutathione S-transferase.
cut01g0060415	no symbol	RasGAP SH3 binding protein rasputin, contains NTF2 and RRM domains.
cut01g0130003	PKR1	V-type ATPase assembly factor PKR1.

* Proteins without any annotations in other yeast genomes.
