# Supplementary material for: Genome and Transcriptome Analysis of the Food-Yeast Candida utilis
Source: PLoS One. 2012 May 18;7(5):e37226. doi: 10.1371/journal.pone.0037226 (PMC3356342; doi:10.1371/journal.pone.0037226)
Supplement: Table S3 — KEGG pathways in which more than 10% of enzymatic genes were classified into high expression genes (uq>10) at log-phase. (PDF) [file pone.0037226.s009.pdf]

Table S3. KEGG pathways in which more than one tenth enzymatic genes were classified into highly expressed genes (uq > 10) at log-phase.

Map ID	Category	Description	The number of genes	The number of highly expressed genes at log-phase	at stationary-phase
map00190	Energy Metabolism	Oxidative phosphorylation	12	8	11
map00524	Biosynthesis of Other Secondary Metabolites	Butirosin and neomycin biosynthesis	16	10	6
map00061	Lipid Metabolism	Fatty acid biosynthesis	20	9	7
map00290	Amino acid Metabolism	Valine, leucine and isoleucine biosynthesis	18	8	6
map00010	Carbohydrate Metabolism	Glycolysis / Gluconeogenesis	44	18	23
map01040	Lipid Metabolism	Biosynthesis of unsaturated fatty acids	15	5	6
*map00970	Translation	Aminoacyl-tRNA biosynthesis	32	9	0
map00250	Amino acid Metabolism	Alanine, aspartate and glutamate metabolism	43	12	11
map00830	Metabolism of Cofactors and Vitamins	Retinol metabolism	18	5	6
*map00906	Metabolism of Terpenoids and Polyketides	Carotenoid biosynthesis	19	5	3
map00710	Energy Metabolism	Carbon fixation in photosynthetic organisms	25	6	10
*map00904	Metabolism of Terpenoids and Polyketides	Diterpenoid biosynthesis	21	5	3
map00940	Biosynthesis of Other Secondary Metabolites	Phenylpropanoid biosynthesis	30	7	8
map00540	Glycan Biosynthesis and Metabolism	Lipopolysaccharide biosynthesis	22	5	6
*map00130	Metabolism of Cofactors and Vitamins	Ubiquinone and other terpenoid-quinone biosynthesis	27	6	4
*map00100	Lipid Metabolism	Steroid biosynthesis	27	6	2
map00051	Carbohydrate Metabolism	Fructose and mannose metabolism	65	12	9
map00620	Carbohydrate Metabolism	Pyruvate metabolism	64	10	14
map00053	Carbohydrate Metabolism	Ascorbate and aldarate metabolism	45	7	6
map00500	Carbohydrate Metabolism	Starch and sucrose metabolism	71	11	11
map00520	Carbohydrate Metabolism	Amino sugar and nucleotide sugar metabolism	95	13	10
map00030	Carbohydrate Metabolism	Pentose phosphate pathway	37	5	8
map00052	Carbohydrate Metabolism	Galactose metabolism	37	5	7
*map00140	Lipid Metabolism	Steroid hormone biosynthesis	38	5	3
map00360	Amino acid Metabolism	Phenylalanine metabolism	58	7	7
map00260	Amino acid Metabolism	Glycine, serine and threonine metabolism	58	7	7
*map00910	Energy Metabolism	Nitrogen metabolism	57	6	5
map00330	Amino acid Metabolism	Arginine and proline metabolism	98	10	12

* Pathways without being selected at stationary-phase.

Table S4. KEGG pathways in which more than one tenth enzymatic genes were classified into highly expressed genes (uq > 10) at stationary-phases.

Map ID	Category	Description	The number of genes	The number of highly expressed genes at log-phase	at stationary-phase
map00190	Energy Metabolism	Oxidative phosphorylation	12	8	11
* map00020	Carbohydrate Metabolism	Citrate cycle (TCA cycle)	22	2	16
* map00720	Energy Metabolism	Reductive carboxylate cycle (CO2 fixation)	13	2	7
map00010	Carbohydrate Metabolism	Glycolysis / Gluconeogenesis	44	18	23
map01040	Lipid Metabolism	Biosynthesis of unsaturated fatty acids	15	5	6
map00710	Energy Metabolism	Carbon fixation in photosynthetic organisms	25	6	10
map00524	Biosynthesis of Other Secondary Metabolites	Butirosin and neomycin biosynthesis	16	10	6
map00061	Lipid Metabolism	Fatty acid biosynthesis	20	9	7
map00290	Amino acid Metabolism	Valine, leucine and isoleucine biosynthesis	18	8	6
map00830	Metabolism of Cofactors and Vitamins	Retinol metabolism	18	5	6
* map00592	Lipid Metabolism	alpha-Linolenic acid metabolism	16	4	5
* map00624	Xenobiotics Biodegradation and Metabolism	1- and 2-Methylnaphthalene degradation	17	2	5
map00540	Glycan Biosynthesis and Metabolism	Lipopolysaccharide biosynthesis	22	5	6
map00940	Biosynthesis of Other Secondary Metabolites	Phenylpropanoid biosynthesis	30	7	8
map00250	Amino acid Metabolism	Alanine, aspartate and glutamate metabolism	43	12	11
* map00640	Carbohydrate Metabolism	Propanoate metabolism	47	4	11
* map00903	Metabolism of Terpenoids and Polyketides	Limonene and pinene degradation	22	2	5
map00620	Carbohydrate Metabolism	Pyruvate metabolism	64	10	14
map00030	Carbohydrate Metabolism	Pentose phosphate pathway	37	5	8
* map00071	Lipid Metabolism	Fatty acid metabolism	29	4	6
map00052	Carbohydrate Metabolism	Galactose metabolism	37	5	7
* map00650	Carbohydrate Metabolism	Butanoate metabolism	53	4	10
* map00630	Carbohydrate Metabolism	Glyoxylate and dicarboxylate metabolism	58	3	9
map00500	Carbohydrate Metabolism	Starch and sucrose metabolism	71	11	11
* map00310	Amino acid Metabolism	Lysine degradation	54	1	8
* map00350	Amino acid Metabolism	Tyrosine metabolism	63	6	9
* map00680	Energy Metabolism	Methane metabolism	35	3	5
map00051	Carbohydrate Metabolism	Fructose and mannose metabolism	65	12	9
map00053	Carbohydrate Metabolism	Ascorbate and aldarate metabolism	45	7	6
map00330	Amino acid Metabolism	Arginine and proline metabolism	98	10	12
map00260	Amino acid Metabolism	Glycine, serine and threonine metabolism	58	7	7
map00360	Amino acid Metabolism	Phenylalanine metabolism	58	7	7
* map00380	Amino acid Metabolism	Tryptophan metabolism	67	6	8
* map00564	Lipid Metabolism	Glycerophospholipid metabolism	52	4	6
map00520	Carbohydrate Metabolism	Amino sugar and nucleotide sugar metabolism	95	13	10

* Pathways without being selected at log-phase.
