# Supplementary material for: Genome and Transcriptome Analysis of the Food-Yeast Candida utilis
Source: PLoS One. 2012 May 18;7(5):e37226. doi: 10.1371/journal.pone.0037226 (PMC3356342; doi:10.1371/journal.pone.0037226)
Supplement: Table S5 — Metabolic pathways defined in the KEGG database in which either S. cerevisiae or C. albicans, or both, lack one or more of the genes in C. utilis within the pathway. (PDF) [file pone.0037226.s011.pdf]

Table S5. Metabolic pathways defined in the KEGG database in which either *S. cerevisiae* or *C. albicans*, or both, lack one or more of the genes in *C.utilis* within the pathway.

Map ID	Description	EC No. not found in *S. cerevisiae*	EC No. not found in *C. albicans*
map01061	Biosynthesis of phenylpropanoids	1.1.1.282, 1.14.11.19, 1.14.11.23, 1.14.11.9	1.1.1.282, 1.14.11.19, 1.14.11.23, 1.14.11.9
map00941	Flavonoid biosynthesis	1.14.11.19, 1.14.11.23, 1.14.11.9	1.14.11.19, 1.14.11.23, 1.14.11.9
map00400	Phenylalanine, tyrosine and tryptophan biosynthesis	1.1.1.24, 1.1.1.282	1.1.1.24, 1.1.1.282
map00640	Propanoate metabolism	6.4.1.3, 4.1.1.41, 4.2.1.17, 3.5.99.7	4.2.1.79, 6.4.1.3, 4.1.1.41, 3.5.99.7
map00280	Valine, leucine and isoleucine degradation	6.4.1.3, 6.4.1.4, 4.2.1.17, 4.2.1.18	6.4.1.3, 6.4.1.4, 4.2.1.18
map01070	Biosynthesis of plant hormones	1.14.11.15, 1.1.1.211, 4.2.1.17	1.1.1.211
map00650	Butanoate metabolism	4.2.1.17, 4.2.1.55	4.2.1.55
map00632	Benzoate degradation via CoA ligation	4.2.1.100, 4.2.1.17	4.2.1.100
map01040	Biosynthesis of unsaturated fatty acids	1.1.1.211, 4.2.1.17	1.1.1.211
map00071	Fatty acid metabolism	1.1.1.211, 4.2.1.17	1.1.1.211
map00062	Fatty acid elongation in mitochondria	1.1.1.211, 4.2.1.17	1.1.1.211
map00410	beta-Alanine metabolism	4.2.1.17	3.5.2.2
map00310	Lysine degradation	4.2.1.17	-
map00380	Tryptophan metabolism	4.2.1.17	-
map00903	Limonene and pinene degradation	4.2.1.17	-
map00930	Caprolactam degradation	4.2.1.17	-
map00592	alpha-Linolenic acid metabolism	4.2.1.17	-
map00281	Geraniol degradation	4.2.1.17	-
map00311	Penicillin and cephalosporin biosynthesis	1.21.3.1, 1.14.20.1, 2.3.1.164	1.14.20.1, 2.3.1.164
map01062	Biosynthesis of terpenoids and steroids	1.14.11.15	-
map00904	Diterpenoid biosynthesis	1.14.11.15	-
map01064	Biosynthesis of alkaloids derived from ornithine, lysine and nicotinic acid	3.5.1.16, 4.2.1.52	3.5.1.16, 4.2.1.52
map00330	Arginine and proline metabolism	3.5.1.16	3.5.1.16
map00300	Lysine biosynthesis	3.5.1.18, 4.2.1.52	3.5.1.18, 4.2.1.52
map00130	Ubiquinone and other terpenoid-quinone biosynthesis	1.13.11.27, 4.1.3.36	4.1.3.36
map00511	Other glycan degradation	3.2.1.96	3.2.1.96
map00230	Purine metabolism	3.6.1.41	3.6.1.41
map00520	Amino sugar and nucleotide sugar metabolism	4.1.3.3	4.1.3.3
map00910	Nitrogen metabolism	1.7.1.4	1.7.1.4
map00053	Ascorbate and aldarate metabolism	4.2.1.41	4.2.1.41
map00270	Cysteine and methionine metabolism	4.4.1.15	4.4.1.15
map00350	Tyrosine metabolism	1.13.11.27	-
map00360	Phenylalanine metabolism	1.13.11.27	-
map00140	Steroid hormone biosynthesis	1.1.1.51	-
map00562	Inositol phosphate metabolism	-	3.1.3.67, 2.7.1.150
map04070	Phosphatidylinositol signaling system	-	3.1.3.67, 2.7.1.150
map00240	Pyrimidine metabolism	-	3.5.2.2
map00983	Drug metabolism - other enzymes	-	3.5.2.2
map00770	Pantothenate and CoA biosynthesis	-	3.5.2.2
map04660	T cell receptor signaling pathway	-	2.7.10.2
